# Training-of-trainers program for community health workers involved in an innovative and community-based intervention against malaria among goldminers in the Guiana shield: a quality and effectiveness evaluation

**DOI:** 10.3389/fpubh.2023.1306432

**Published:** 2024-01-08

**Authors:** Carlotta Carboni, Irene Jimeno Maroto, Muriel Galindo, Lorraine Plessis, Yann Lambert, Teddy Bardon, Stephen Vreden, Martha Suárez-Mutis, Jane Miller Bordalo, Maylis Douine, Alice Sanna

**Affiliations:** ^1^Département Recherche, Innovation et Santé Publique, Centre d’Investigation Clinique Antilles-Guyane (Inserm 1424), Centre Hospitalier de Cayenne, Cayenne, French Guiana; ^2^Foundation for the Advancement of Scientific Research in Suriname (SWOS), Paramaribo, Suriname; ^3^Laboratório de Doenças Parasitárias, Instituto Oswaldo Cruz, Fiocruz, Rio de Janeiro, Brazil; ^4^DPAC Fronteira, Oiapoque, Brazil

**Keywords:** training-of-trainers, training programs, community health workers, capacity building, malaria, goldminers, Amazon

## Abstract

**Introduction:**

An innovative and community-based intervention is implemented in the Guiana Shield to eliminate malaria among people involved in artisanal and small-scale gold mining. The intervention consists of the distribution of malaria self-management kits to goldminers and the presumptive treatment for individuals at risk of carrying *Plasmodium vivax* hypnozoites. The intervention is possible owing to community health workers (CHWs) who are previously trained to master all intervention procedures, including health education activities and goldmining training. This study aimed to evaluate the training program provided to CHWs in terms of quality and effectiveness.

**Methods:**

A training-of-trainers program for CHWs has been developed based on the CDC framework. A mixed-method case study was implemented in two steps between February and March 2023. The evaluation was based on a knowledge survey, satisfaction test, observations, and semi-structured interviews. Quantitative and qualitative data were analyzed and triangulated.

**Results:**

A total of 20 CHWs participated in the training and the first-step evaluation. For the second step, four semi-structured interviews were conducted. The Qualitative data showed that group dynamics and adaptations were central elements of a high-quality training program. Quantitative analysis found that CHWs’ satisfaction was elevated (> 4/5 overall), especially regarding format and learning results. Improvements in knowledge level demonstrated good effectiveness (pre-training vs. post-training, *p* < 0.05). Nevertheless, some difficulties persisted regarding tasks of the intervention procedure, such as informed consent and smartphone application procedures (with an inaccuracy rate of 29.2% and 16.7%, respectively). Further on-the-job training permitted to address these issues. The project team’s previous experience and the Guiana Shield countries’ commitment to the WHO-E-2025-initiative were identified as levers for the quality of the training, while the complexity of the project context was a challenge.

**Discussion:**

High-quality, effective, and appropriate training programs are required for effective and sustainable interventions involving CHW profiles. Training design is a crucial point to address to accomplish quality and effectiveness. The training-of-trainers model has been shown to allow a high level of satisfaction, good learning results, and satisfactory implementation in the field. Initial and continuing training is an indispensable continuum to sustain good practices in the field and CHWs’ motivation. Training evaluation permits standardizing methods and facilitates transferability.

## 1 Introduction

Since 2000, the malaria burden has considerably decreased in the Americas; however, it is still endemic in the Guiana Shield (1–3). The fight against malaria in the Guiana Shield is challenging as the major malaria reservoir in the area is represented by a mobile and hard-to-reach population (4, 5). This population is mainly represented by Brazilians (95–98%) involved in artisanal and small-scale gold mining (ASGM) in the Amazonian rainforest, where they live in a situation of vulnerability with reduced access to the healthcare system (6, 7). At least 10,000 individuals are involved in ASGM in French Guiana, and the prevalence of malaria in this population was estimated to reach 46.8% in some areas of this country in 2015 (6, 7).

Despite the complexity of reaching populations suffering from social inequality, strategies have been developed to provide healthcare access (8). Indeed, at a global scale, community-based interventions play a fundamental role in improving population health and increasing access to healthcare and services (9, 10). Worldwide, a range of effective interventions involving community health workers (CHWs) exists, especially to fight malaria (11, 12). In malaria elimination programs, CHWs are often involved in case management, malaria surveillance, diagnosis, treatment, prevention, and health promotion activities (13).

A complex, innovative, and community-based interventional research was implemented during the period 2018–2020 as a result of a scientific partnership established between Brazil, French Guiana, and Suriname, focusing on populations involved in ASGM in the Guiana Shield in situations of extreme isolation from health services (14). The aim of this research was the evaluation of a strategy that provides access to diagnosis and treatment through the distribution of kits (called “malakits”), associated with training to use it correctly (15). The malakit was composed of self-testing kits with rapid diagnostic tests (RDTs) and self-treatment kits to be used in case of a positive result. This strategy was evaluated as effective and acceptable by the community and has been scaled up in Suriname (16–18).

In recent years, a decrease in malaria prevalence and the inversion of the proportion of *Plasmodium falciparum* and *Plasmodium vivax* which is typical of malaria elimination phases has been observed (19). Specifically, this proportion reached, respectively, 91% vs. 7% in French Guiana by the first semester of 2022 (19). Thus, in 2023, Curema, another interventional research, has started. This new research expands the foundations of the Malakit project to also include and evaluate the radical cure of *P. vivax* (20, 21). Curema intervention consists of two modules: the “malakit” module and the “radical cure” module. The latter aims to prevent relapses and thus reduce the number of human hosts capable of transmitting the parasite; asymptomatic individuals at risk of carrying *P. vivax* hypnozoites are offered presumptive treatment with chloroquine and 8-aminoquinoline (primaquine or tafenoquine). The Curema intervention is offered at inclusion sites—malaria clinics, hotels, or rooms rented specifically for the project—located in Brazil and Suriname in resting and logistical places across the border in French Guiana, regularly frequented by people involved in ASGM in French Guiana.

**Figure 1:**
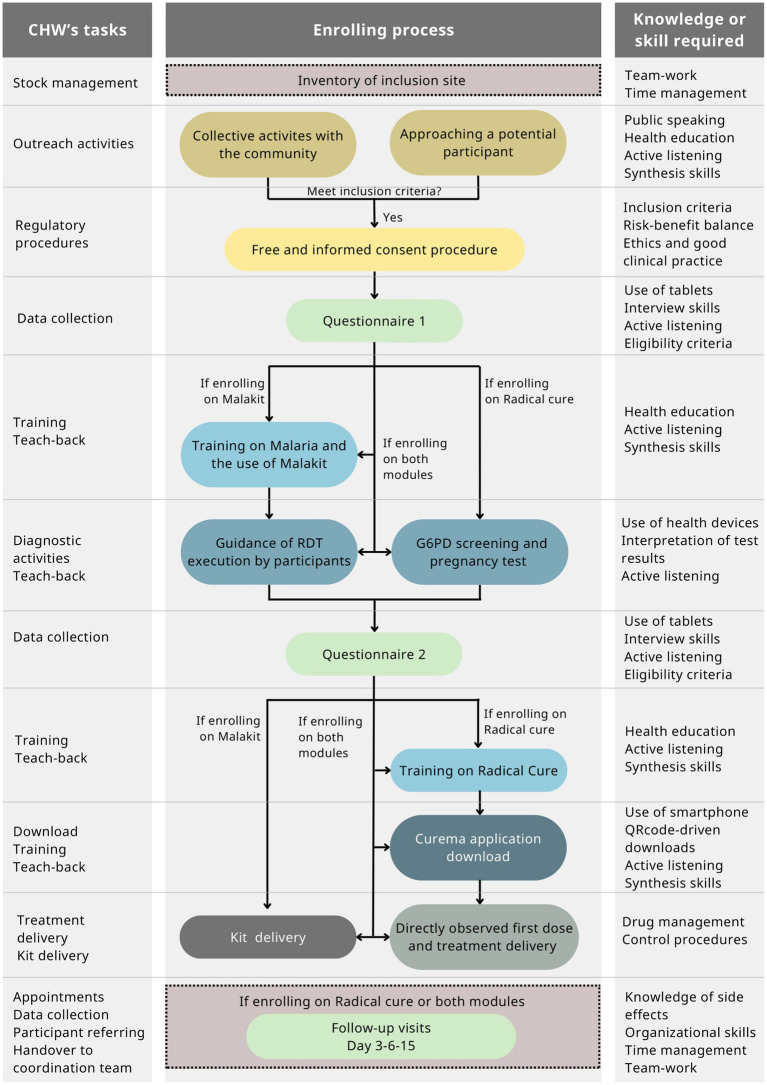
CHW tasks over the Curema enrolling process and knowledge/skills associated with.

The intervention is made possible by CHWs, specifically recruited for the Curema project, who work in pairs on the field at the inclusion sites. As part of the Curema project, CHWs are responsible for enrolling participants and providing health education to participants followed by individual training during the inclusion process (Figure 1). Furthermore, CHWs carry out collective outreach activities on malaria in the community. CHWs are selected from the same community as the target population: They know the language, lifestyles, and culture of this population. As a result, they can reach a larger number of individuals. In fact, because of the illegal administrative situation and activities of the majority of people involved in ASGM, this population is reticent toward outsiders; thus, a community-based intervention with the involvement of CHWs helped gain their trust.

Before the launch of the project, the CHWs participated in a training program that enabled them to take part in a bidirectional working space, to master health research procedures, to present information effectively, to lead activities that reinforce participants’ learning, and to collaborate on the design of working tools. Moreover, they were supported during the gradual launch of the project in the field and regularly supervised during the whole project implementation.

Despite the large integration of CHWs in public health interventions, little is known about best practices for their training. Training-of-trainers (ToT) programs are often used for capacity building in the health workforce. ToT is also known to be a predictor of sustainability. In fact, this methodology allows a training cascade that can cheaply and exponentially have effects on a larger amount of individuals than the initial trainees: Small groups of trainees, in turn becoming trainers, can carry out knowledge transfer from one to another (22).

To ensure effective CHW activities, it seems vital to provide good training and continuous professional development through supervision. Nevertheless, literature is often lacking evidence on the implementation and evaluation of training programs. The implementation of ToT programs in the field of infectious diseases and cross-border contexts is not well reported in the literature (23). Several theoretical frameworks have been developed for evaluating this process, but the introduction of standardized evaluation methods in the context of training evaluations is increasingly necessary (22–25).

Therefore, it seems crucial to share findings and experiences in this domain with the scientific community to facilitate the transferability of successful efforts.

This present article aims to illustrate the results of an evaluation of the quality and effectiveness of the training program offered to CHWs involved in the Curema project.

## 2 Materials and methods

### 2.1 Description of the Curema training program

#### 2.1.1 CHWs’ recruitment

CHWs were recruited to work in pairs at the Curema project inclusion sites located in Brazil (2 sites) and Suriname (5 sites). The recruitment process was conducted separately by local partners for Brazilian and Surinamese Curema project inclusion sites with the support of the project’s promoter, the Hospital of Cayenne, French Guiana. Their profile is similar to CHWs involved in malaria intervention in other countries (26, 27). CHWs’ recruitment criteria were: being over 18 years of age, belonging to the community involved in ASGM or knowing it closely, speaking fluent Portuguese, being able to use information technology tools (tablets, smartphones, etc.), and living in or being prepared to move to one of the project locations for inclusion sites.

The CHWs working at the Brazilian inclusion sites were employed by the NGO DPAC Fronteira (*Desenvolvimento, Prevencao, Acompanhamento e Cooperacao de Fronteiras*; Development, Prevention, Support, and Cooperation on Borders) and were only involved in activities linked to the Curema project. In fact, CHWs working for DPAC Fronteira were specifically recruited for the Curema project; thus, they did not have any specific role in malaria control or malaria case management in the context of the Brazilian national health system. DPAC Fronteira selected CHWs before they underwent the training program.

At the Surinamese inclusion sites, the CHWs were employed by the Surinamese research foundation SWOS for the Curema project (half-time) and the Surinamese National Malaria Elimination Programme (NMEP) of the Ministry of Health for the second half-time as “malaria service deliverer” (MSD). The MSD role involved malaria case management (diagnosis, treatment, notification, and investigation) and distributing mosquito nets in remote areas related to ASGM (28). Indeed, in Suriname, CHWs had specific roles in malaria control as part of the national strategy for malaria elimination in addition to tasks related to the Curema project. CHWs for Suriname inclusion sites underwent a two-step recruitment process; consequently, a larger number of potential CHWs were invited to attend the training program. Most CHWs invited to attend the Curema training program already functioned as MSDs in the mining fields. It was made clear to all trainees that participating in the training was no guarantee for being selected to work in the Curema project. Final CHWs were selected after the end of the initial training, just before the launch of the project in the field.

Before the Curema training program, CHWs undertook basic training sessions separately as a pre-work. In Brazil, a basic 1-day training on malaria was provided by one of the authors (Fiocruz), and a training on NGO work and community mediation was provided by DPAC Fronteira, an NGO. In Suriname, CHWs received initial and refresher training on malaria prevention, diagnosis, and treatment techniques by NMEP.

#### 2.1.2 Design and development of the training program

**Figure 2:**
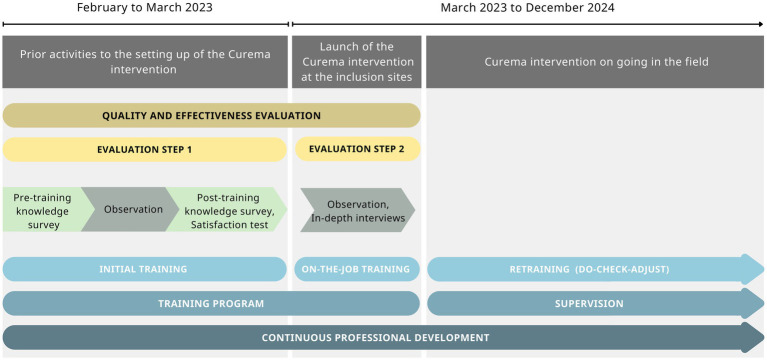
Timeline and flowchart of the training design and training evaluation over the Curema intervention phases.

To make CHWs able to master all the procedures and tasks they need to carry out, the Curema training program was designed using a training-of-trainers (ToT) model with two components: an initial training and an on-the-job training (Figure 2).

Adult learning principles were applied to design the training program focusing on practice-based activities to be appropriate for CHWs’ needs and to allow them to practice (29). To be more effective and to reach all the audience, varied activities and methods were implemented, mobilizing cognitive, affective, and psychomotor learning principles and the visual, auditory, and kinesthetic (VAK) learning model (30–32). Moreover, the training program was tailored and centered on CHWs. Therefore, the training program was conducted in Portuguese using learning tools targeting an audience with a low level of scholarity.

All partners and organizations involved in the Curema project participated in the training program that was rich in contributions from all staff and professionals with different experiences and expertise. Trainers used a horizontal approach to make CHWs feel respected and to make the learning environment safe and supportive. To be more responsive to the needs of the CHWs and the community in the field, a bidirectional approach was used, and CHWs were asked to collaborate in the design of operating tools.

The initial training component took place in Paramaribo, Suriname, jointly by the entire team of CHWs both from Suriname and Brazil. Considering the type and number of topics to be addressed, the initial training was developed for 3 weeks to allow enough time to ensure an effective knowledge transfer, an exhaustive practice of skills and new procedures, an appropriate adaptation of tools, and a good team and capacity building.

The on-the-job training component took place at each inclusion site and lasted 2–3 days. It aimed to support CHWs, while the project was gradually rolled out in the field and the first inclusions were carried out.

The components included in the ToT program, as the Centers for Disease Control and Prevention recommend, are shown in Table 1 (33).

**Table 1:** ToT components included in the Curema training program.

ToT component	Pre-assessment	Pre-work	Training agenda	Facilitation manual	Modeling of skills and tasks
**Aim**	Identifying pre-training knowledge and skills of the CHW to determine training needs	Providing CHWs with the knowledge and background needed before the training program	Providing some degree of control over what, when, and how CHWs will learn during the training program.	Organizing tools and improving the capacity of CHWs to transfer knowledge	Enabling CHWs to form a clear idea of how to carry out a task by showing them how to perform a skill while describing each step
**Initial training**	Pre-training knowledge survey	Topics: basic knowledge about malaria (diagnosis and treatment), epidemiology in the Guiana Shield, and malaria control strategies Pre-work training sessions took place separately for the CHWs working on the inclusion sites located in Brazil or Suriname, due to different recruitment frameworks. Those sessions were provided, respectively, by two different organizations due to the legitimacy of the respective countries	Provided on the first day of the training	Audio-visual tools (i.e., a step-by-step tutorial about how to perform a Malakit RDT) were uploaded to tablets Visual sheets for explanation support were put together in a CHW binder Games and teach-back cards were also provided as teaching tools	Following training units: Audio-visual tutorial for Malakit RDT and for G6PD test; Information Education and Communication (IEC) strategies and tools use; Inclusion process simulation role-play; Informative note and informed consent procedure role-play
**On-the-job training**	Post-initial-training knowledge survey Feedback from the trainers regarding the initial training program outcomes	Initial training	Not applicable	The same tools and CHW blinder with the addition of some updated sheets	Not applicable

ToT component	Adults learning principles	Skill practice	Feedback	Action planning	Planned follow-up support
**Aim**	Diversifying activities based on the VAK Model and Bloom’s Taxonomy, enhancing experience, designing a problem-centered training program to reach CHWs, and making the training effective	Providing the opportunity for the practice of selected training activities or contents.	Collecting CHWs’ feedback regarding the learning process to be adjusted.	Taking CHWs through the process of creating or adjusting tools and practices for the intervention to succeed.	Strengthening the knowledge and skills of CHWs, so they can be applied effectively.
**Initial training**	Implementation of games, practical simulations, video making, and a variety of activities with movements needed Activities in small working groups composed of heterogeneous profiles for skills and personalities	Practice of: data collection, tablet use, G6PD test execution, Malakit RDT execution monitoring, participants and community training, health education, public speaking, inclusion process simulations, installation of Curema application for smartphone	Daily debriefing at the beginning of the day regarding previous content, doubts, and difficulties. Use of a checklist to help complete the whole inclusion process Satisfaction assessment and discussion group on the last day	Information Note video – for the signature of the ICF (Informed Consent Form) Feedback about Questionnaire	Continuous professional development through ongoing training for the intervention launch on the inclusion sites.
**On-the-job training**	Directly linked to on-site work and problem-solving	Inclusion process and community outreach activities	Debriefing together at the end of the inclusion process (either real process or simulations)	CHWs’ initiatives encouraged (group of discussions, projection of promotional videos, marauding and outreach activities, etc.)	Continuous professional development and targeted follow-up: (i) regular supervision visits (ii) remote support via a specific WhatsApp© group

### 2.2 Evaluation of the quality and effectiveness of the training program

#### 2.2.1 Framework

Evaluation was carried out for the quality and effectiveness of training.

Quality was defined by the International Standard Vocabulary as “all the characteristics of an entity that influence its ability to satisfy expressed and implicit needs” (34). In this context, authors referred to “needs” as the outcomes to be achieved by CHWs through the training program.

Effectiveness corresponded to the extent to which the training outcomes are achieved. According to Kirkpatrick’s theoretical framework, effectiveness is assessed at four levels: reaction, learning, behavior, and system (35).

The elements to be evaluated were defined as follows (25):

• Quality assessment

o Context: external factors that can influence the training program, both as obstacles and levers, and which cannot be controlled by the training deliverers.
o Input: internal conditions under which the training program takes place (e.g., methods used by trainers, CHWs’ prior knowledge, and group dynamism).
o Process: the training delivery process, including the adaptation, dose delivered, and CHWs reached.

• Effectiveness assessment

o Reaction: level of CHWs’ satisfaction in terms of dose and content.
o Learning: changes in the level of knowledge, know-how, and interpersonal skills.
o Behavior: level of concordance of CHWs’ practice with the reference (Curema protocol).
o System: impact of CHWs’ practice on community health education.

The item regarding system impact has not been evaluated at this stage of Curema intervention.

#### 2.2.2 Methods

The design of the training evaluation consisted of a mixed-method case study, executed in two steps as shown in Figure 2 first, during the initial training in Paramaribo, a classroom environment was created, and second, on the occasion of on-the-job training, provided during the launch of the intervention on the field. Quantitative and qualitative methods nurture the first step, while the second step is nurtured only by the qualitative method. The quantitative component provided computable data on satisfaction, knowledge improvement, and practice, while the qualitative component enabled giving context and an in-depth explanation of the quantitative results.

The inclusion criterion for the evaluation of step 1 was being a CHW attending the initial training and agreeing to participate. The study location for this step was the Clevia Park training center in Paramaribo, Suriname. However, the CHWs remotely undertook the online follow-up knowledge survey. Step 1 took place from February to March 2023. Step 1 consisted of knowledge surveys (pre-training, immediate post-training, and 1-month follow-up), a satisfaction test, and observations (informal conversations with participants and trainers, participants observation, systematic inclusion process monitoring through a checklist, documentary research, and discussion groups).

The inclusion criterion for the evaluation of step 2 was being a CHW assigned to the Curema project inclusion sites launched in March 2023 and agreeing to participate. The study locations for step 2 were the Curema project inclusion sites located in Ronaldo and Yawpassi, Suriname. The study took place in March 2023. Step 2 consisted of observations (informal conversations with participants and trainers, participants observation) and semi-structured interviews.

Quantitative data were collected through knowledge surveys, a satisfaction test, and observations (checklist tool), while qualitative data were collected through observations and semi-structured interviews and analyzed, as described in Table 2.

**Table 2:** Methodology used to evaluate the quality and effectiveness of the Curema training program.

Evaluation method	Framework component	Collection tool	Inclusion criteria	Temporal aspects	Analysis	Software
**Knowledge survey**	Learning; Input	Basic and advanced multiple-choice and true-false questions	All CHWs attending the training program who agreed to undergo the knowledge survey (*N* = 18 at t_0_; *N* = 16 at t_1_; *N* = 15 at t_2_)	Evaluation step 1: Pre-training (t_0_), immediate post-training (t_1_) and 1-month follow-up (t_2_) questionnaires	Mann–Whitney–Wilcoxon U test to compare average scores; Wilcoxon signed ranks test to compare average scores only for CHWs who answered the three tests; Bivariate analysis of the following variables: age, gender, recruitment country, mother tongue, previous collaboration, retained after training	Microsoft® Excel® for Microsoft 365 MSO (Version 2,304 Build 16.0.16327.20200) Stata® (Version 14.2 Copyright 1985–2015 StataCorp LLC).
**Observation (direct and indirect)**	Context; input; process; reaction; learning; behavior	Observation guide to collect: Informal conversations with participants and trainers; Participant observation; Systematic inclusion process monitoring through a checklist; Documentary research; Discussion groups	Evaluation step 1: All CHWs attending the initial training who agreed to participate in the observation (*N* = 20) Evaluation step 2: Convenience sampling depending on the inclusion site to be launched. CHWs assigned to the predetermined inclusion site who agreed to participate in the observation (*N* = 4)	Evaluation step 1: During the initial training Evaluation step 2: During on-the-job training	Inductive content analysis; Descriptive analysis for quantitative data	ATLAS.ti® (Version 23.1.2.0, license L-685-998). Microsoft® Excel® for Microsoft 365 MSO (Version 2,304 Build 16.0.16327.20200)
**Satisfaction test**	Reaction	Single-choice and a 5-point-Likert-scale test (anonymous test) to explore opinions on methodology, relevance, clarity, trainers support, practice, facilitation manual, group dynamics, and logistic	All CHWs attending the initial training who accepted to fill in the satisfaction test (*N* = 18)	Evaluation step 1: At the end of the initial training	Descriptive analysis	Mentimeter® (Version 3.2.7, 26-08-2021)
**Semi-structured interviews**	Input; process; learning; reaction; behavior	CHWs interviews: an interview guide was developed based on preliminary results of the observations	Evaluation step 2: Convenience sampling depending on the inclusion site to be launched. CHWs assigned to the predetermined inclusion site who agreed to be interviewed (*N* = 4)	Evaluation step 2: During on-the-job training (4 h 38′ 18″)	Description analysis on the verbatim (Brazilian Portuguese) mainly deductive with inductive elements	ATLAS.ti® (Version 23.1.2.0, license L-685-998).

The analysis was conducted using a theoretical framework conceptualized for the evaluation of training programs for the control of infectious diseases in cross-border contexts by the Centre for Infectious Disease Control, the National Institute for Public Health of Health of The Netherlands, and the EU Healthy Gateways Joint Action (25). Different tools and collection methodologies have been used to assess each component of the framework, as reported in Table 2.

#### 2.2.3 Ethics

The Curema protocol was approved by the ethics committees of the countries involved in the project (Brazil: CONEP:5,507,241 and Suriname: CMWO05/22). The evaluation of the quality and effectiveness of CHWs’ training program is part of the secondary objectives of the Curema protocol.

According to the article R1121-1 of the French Public Health Code, the regulatory classification of this evaluation is a “research not involving the human being” (outside the scope of the *Loi Jardé* or *RNIPH*) because it “aims to evaluate the methods of practice of healthcare health professionals or teaching practices in the field of health” (36). A collective oral information was reached out, and participants’ non-opposition was collected. For semi-structured interviews, consent was requested.

The evaluation was conducted under the principles of the Declaration of Helsinki. Data collected were recorded in accordance with French law and the European Union General Data Protection Regulation. Data collected, field notes, and transcribed recordings were anonymized and stored on a secure server.

## 3 Results

The initial training took place between February 13, 2023 and March 3, 2023. On-the-job training started with the launch of the intervention at the first inclusion site on March 13, 2023 and lasted 2–3 days per site. The evaluation was carried out between February 13, 2023 and April 3, 2023.

### 3.1 CHWs’ profile

A total of 20 CHWs were invited for the training program, 14 (70%) from Suriname employer organizations and 6 (30%) from Brazil employer organization. Disparities of participants between Suriname and Brazil were due to the recruitment framework (final selection before vs. after the training program) and the number of Curema inclusion sites in Brazil vs. Suriname (2 vs. 5). The characteristics of the trainees are summarized in Table 3. The majority of CHWs belonged to female sex, were over 40 years old, and spoke Portuguese as their mother tongue. Five CHWs had Spanish as their mother tongue but could proficiently speak and understand Portuguese. In total, 20% of CHWs had previously worked in research. Of the 20 CHWs, four took part in individual semi-structured interviews during evaluation step 2.

**Table 3:** Characteristics of CHWs who attended the initial training program in Paramaribo and who participated in semi-structured interviews.

CHWs characteristics	Involved in the initial training in Paramaribo (*N* = 20)		Involved in semi-structured interviews (*N* = 4)	
	Percentage (%)	Number (*n*)	Percentage (%)	Number (*n*)
Sex				
Male Female	20.0 80.0	4 16	25.0 75.0	1 3
Age (years)				
18–40 >40	20.0 80.0	4 16	25.0 75.0	1 3
Country of the employer organization				
Brazil Suriname	30.0 70.0	6 14	0.0 100.0	0 4
Mother tongue				
Portuguese Spanish	75.0 25.0	15 5	100.0 0.0	4 0
Previous collaboration with the employer organization				
Yes	50.0	10	100.0	4
Previous work experience in research				
Yes	20.0	4	0.0	0
Belonging to ASGM-related community				
Yes	70.0	14	100.0	4
Retained after training				
Yes	75.0	15	100.0	4

CHW profiles were different in Brazil and Suriname because of different recruitment criteria and frameworks. For instance, 33.3% of CHWs from Brazil has already worked in research-related field, although only 14.3% of Surinamese has worked in the research-related field. In addition, 100% of CHWs from Brazil employer organization were recruited, whereas 64.3% from Suriname employer organization were recruited.

### 3.2 Results of the quality evaluation

#### 3.2.1 Contextual levers and difficulties

Contextual elements have been identified through indirect observation, particularly through documentary research.

Two elements were identified as levers for the training. The first is the political, economic, and intellectual environment in which the Curema project—and consequently the training program—navigates. In fact, Suriname and French Guiana have committed to eliminate malaria from their territories by 2025 (World Health Organization’s E-2025 initiative) and Brazil by 2035 (1, 37, 38). There is no doubt that this commitment ensured good conditions for the start of the Curema intervention and its training program. Second, previous lessons learned during the Malakit project by our team in training the same community in a similar context and partners’ experiences were precious in guiding the design and development of the ToT program, particularly regarding the methodology, the time, and the appropriate tools for the audience (39).

On the other hand, the complexity of the Curema project also influenced the training because of a large number of interacting components that are sometimes difficult to align for the training program development (40). In fact, Curema is an international project that means several organizational levels, country-specific features for implementation on the field, communication in different foreign languages depending on the actors, and logistical issues linked to differences in bureaucracy and regulatory requirements between countries (41). Moreover, it was important to consider that the number and level of articulations of the activities required by CHWs complexified the training program design. Finally, differences in recruitment and employment frameworks made CHW group composition heterogeneous: Previous baseline knowledge was dissimilar due to previous experiences and to a pre-work organized separately for Brazil and Suriname.

#### 3.2.2 The importance of group dynamics

Among the CHWs who attended the training program, four (20.0%) had already worked in the research field and ten (50%) had already collaborated with one of the three employer organizations involved in the Curema project. We observed that, for these CHWs, prior knowledge of certain procedures increased mastery of the subject and reduced the time needed to learn. In addition, owing to a participatory approach and to the inclusion in the schedule of practical sessions of pair and group work, peer education dynamics took place. The attention to the choice of the classroom and the setting is also decisive in the generation of these dynamics. Thus, these CHWs became a resource for supporting training. However, certain disadvantages were also identified such as the transfer of some erroneous automatisms already adopted in their practice. Feedback activities allowed to readjust practices.

*“Teamwork is always very good to bring together (experiences) […] often Brazil works in one way. We work in another way […] and this interaction helps us a lot, I learn from you and you learn from me, and* vice versa*.” (Interview verbatim, CHW from Suriname).*

The observed interpersonal relationships among CHWs had a strong influence on group dynamics for learning, active participation in training, and engagement in the project. Because of the different recruitment framework, at the beginning of the training program CHWs appeared to form two distinct groups. Based on team building and adult learning principles, more collaborative activities were implemented to facilitate exchanges and integration. Some spontaneous team-building initiatives were also pointed out.

*“Afterwards, the group work was done, which […] allowed people to feel more in tune with each other and communication improved […] which was fun and at the same time a joint learning process, it was very good.” (Interview verbatim, CHW from Suriname).*

#### 3.2.3 A flexible and evolutive process

The training was delivered in 105 hours over 3 consecutive weeks (Table 1). The training program was designed to be part of a continuous professional development approach centered on CHWs. Thus, it was flexible and evolutive. Numerous adaptations were made to ensure constant improvement of CHW knowledge and practice and to perfect training (schedule, activities, and tools) and project elements. Indeed, many of those elements had already been co-designed through community involvement. The bidirectional working space during the training sessions enabled to adapt the vocabulary and content of the inclusion questionnaire, add supplementary material in the facilitation manual, and make a video for the informed consent procedure. As they reported during informal conversations and semi-structured interviews, CHWs greatly appreciated the opportunity to be part of this evolution process.

*“I read the informative note […] But thanks to the video activity, I read and memorized (the informative note). What I had to say in the video, that’s what I learned, I do not forget it.” (Interview verbatim, CHW from Suriname).*

### 3.3 Results of the effectiveness evaluation

#### 3.3.1 Reaction: participants’ satisfaction

The reaction has been assessed through participant observations, informal conversations with participants, the group discussion, satisfaction test, and semi-structured interviews.

Overall, CHWs affirmed to be very satisfied with the training. The methodology used for the training was particularly appreciated by CHWs with reference to activities using a kinesthetic learning model, practice-based activities, group work, and a bidirectional approach. All items explored on a scale of 1–5, ranging from “not at all satisfied” to “completely satisfied,” were ranked over 4. Satisfaction is shown to be directly linked to learning:

*“It was fun, creative, and useful for memorizing, because you remember [*what you learn using these techniques*], is not it? We do not forget. The games were very good” (Interview verbatim, CHW from Suriname).*

Indeed, 61.1% of the 18 CHWs answering the satisfaction test declared had reached “optimum mastery,” the 27.8% reported “sufficient mastery but needed support during the launch in the field”; and only one person felt “insufficient mastery,” asking for “more time for training.”

CHWs were observed to be satisfied also during the on-the-job training. In fact, some of them stated that the sessions provided the knowledge and practice needed to start the job in the field.

#### 3.3.2 Learning: evaluation of knowledge improvement

Learning has been evaluated through knowledge surveys and observations (particularly using the systematic inclusion process monitoring checklist). Self-assessment of achieved learning goals also provided data for this section through informal conversations and semi-structured interviews.

**Figure 3:**
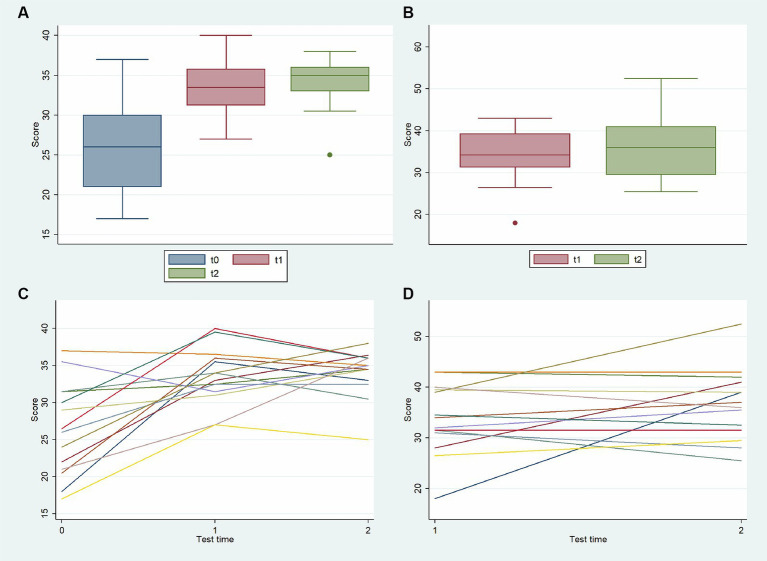
Evolution over time in the knowledge survey score for basic knowledge (at t_0_, t_1_, and t_2_) among all CHWs **(A)**, for advanced knowledge (at t_1_, and t_2_) among all CHWs **(B)**, for basic knowledge (at t_0_, t_1_, and t_2_) per CHWs **(C)**, and for advanced knowledge (at t_1_, and t_2_) per CHWs **(D)**.

The knowledge survey evaluation found a knowledge improvement with a significant increase in the average scores of the test between pre- and post-surveys (basic knowledge) with a *p*-value of <0.05 both for test scores at t_0_−t_1_ and t_0_−t_2_ (Figure 3). On the contrary, differences in scores between tests at t_1_ and t_2_ were not significant both for basic and for advanced knowledge (*p* = 0.26 and *p* = 0.68, respectively). The same trend was shown for analyzing individual scores’ increase for CHWs who underwent the knowledge survey at t_0_, t_1_, and t_2_. The bivariate analysis (Table 4) for age, sex, recruitment country, mother tongue, and previous collaboration did not show any differences in these categories, apart from a better basic knowledge in the t_2_ test among those with previous experience in the research field (*p* < 0.05).

**Table 4:** Knowledge scores of CHWs at pre-training (t_0_), immediate post-training (t_1_), and 1-month follow-up (t_2_) questionnaires, Curema Project, 2023.

CHWs characteristics	Score averages					*p*				
	t_0_ (*N* = 18)	t_1_ (*N* = 16)		t_2_ (*N* = 15)		t_0_	t_1_		t_2_	
	Basic	Basic	Advanced	Basic	Advanced	Basic	Basic	Advanced	Basic	Advanced
Sex										
Male Female	23.75 26.65	31.83 33.81	35.25 33.99	35.00 34.15	32.50 31.62	0.395	0.543	0.742	0.946	0.191
Age (years)										
18–40 >40	20.75 26.67	32.00 33.92	35.25 34.18	36.75 33.50	36.50 36.00	0.156	0.465	0.864	0.362	0.865
Country of the employer organization										
Brazil Suriname	28.08 24.82	34.25 32.95	33.75 34.71	35.58 33.55	34.00 37.44	0.208	0.703	0.096	0.871	0.443
Mother tongue										
Portuguese Spanish	25.79 26.83	32.54 36.13	34.07 36.00	33.83 35.75	36.69 32.00	0.659	0.201	0.304	0.627	0.395
Previous work experience in research										
Yes No	25.25 27.00	34.75 32.13	35.66 32.33	33.69 34.94	36.94 34.75	0.558	0.113	**<0.05**	0.916	0.680

Bold value indicates statistical significance *p* < 0.05.

A total of 24 inclusion simulations have been studied during the evaluation step 1. Systematic inclusion process monitoring through the checklist pointed out that two-thirds of the observed inclusion simulations (*n* = 16) contained inaccuracies in the step-by-step process. Nevertheless, inaccuracies often concerned the same steps. The highest rate of inaccuracy was found for the explanation of the patient information note for the informed consent procedure, which was inaccurate in 29.2% of the observed inclusion simulations, followed by the process of installing the Curema smartphone application, which had an inaccuracy rate of 16.7%.

These results are in line with the results of the qualitative analyses based on informal conversations and interviews with CHWs who also reported reasons why those two steps were difficult in their opinions. First, the information note, using scientific terms and constructions, was dense with complex concepts and difficult to be understood by people with a poor level of scholarity.

*“What really frightened us, I can say for myself and several other people, was the informative note […] What really complicates things for me is the informative note.” (Interview verbatim, CHW from Suriname).*

Second, the Curema smartphone application was not sufficiently explored during the training program. Indeed, at that time, some technical developments of the application were still in progress. Thus, the final version and procedure to use it were not integrally exposed during the initial training.

*“(The Curema application procedure), we have only seen it one day, have not we? We have not tested it much yet.” (Interview verbatim, CHW from Suriname).*

#### 3.3.3 Behavior: from the training to the field

Behavior was assessed during evaluation step 2 using observation techniques and semi-structured interviews.

During the observed launch at the Curema inclusion sites, the level of concordance of CHWs’ practices with the Curema standard procedures was adequate even if, during on-the-job training, further support by trainers was needed to guide CHWs toward autonomy. Moreover, CHWs expressed the need for more time and fieldwork to master the step-by-step process and re-elaborate concepts for an effective knowledge transfer to the community.

*“I think everything went well. Now it’s just a matter of practice, in my opinion.” (Informal conversation, CHW from Brazil).*

Constructive professional relationships, good teamwork, and a sense of belonging to a common cause were also pointed out by CHWs as factors able to influence behavior in the field, leading to engagement and proactive participation in the intervention.

*“If you do not feel good in the team, this can have an influence on your motivation, for example, in the way the work is run.” (Interview verbatim, CHW from Suriname).*

## 4 Discussion

High-quality, effective, and appropriate training is required for effective and sustainable intervention involving CHWs profiles. This involves a great deal of effort in development, implementation, and evaluation. Moreover,this study emphasizes that the effectiveness of a training program depends on its quality. The ToT model has been shown to allow a high level of satisfaction, good learning results, and satisfactory implementation in the field.

### 4.1 Training design: a crucial step

Developing a high-quality training program demands careful considerations of the learning methods and modules to be designed. We designed the training program on a ToT model based on adult learning principles. Consequently, the training program contained ample interactive sessions including group discussions, role plays, practical activities, and simulations. This methodology is considered more effective in comparison with traditional teaching approaches, especially among CHWs with a poor level of scholarity (9, 42). This was also confirmed in our results. Despite complexities, we can affirm that the quality of the training was good and met the needs for the implementation of the Curema intervention in the field. The significant increase in pre-post scores obtained at the knowledge survey suggests the achievement of theoretical concepts. Moreover, the ample panel of activities contributed to an increase in satisfaction levels, which is consistent with the existing literature (25).

A crucial point to be discussed is adaptations. During the training program, several adaptations were necessary to be adopted both for the training program and for the intervention on the field. We could even say that one of the aims of the Curema training was to enable these adaptations to be made thanks to the bidirectional space setup with the CHWs. Despite the balance between adaptation and fidelity in implementation science is still a topic of debate (43–45), several examples in the literature described helpful adaptations: Miller et al. adapted the terminology of their intervention to reduce the possibility of misinterpretation of their study population; while Holtrop et al. described that adaptations in training programs or in recruitment were due to outside forces or made to improve feasibility or engagement (46, 47). A review by Stirman et al. identified most common reasons for intervention adaptation were to address language, cultural differences, literacy, or situational constraints (48). In our experience, we can argue that adaptive aptitude, embedded in a community-based approach, may have enabled our intervention to be more appropriate, effective, and sustainable by fostering the bidirectional transfer of knowledge, the empowerment of CHWs as a central actor of the intervention, and their engagement in the Curema project.

To be appropriate, trainings of the CHWs should take place in their work environment to practice in a real-life situation (49, 50). Our quantitative and qualitative results suggested that, although many simulations were carried out during the initial training, real practice in the field during on-the-job training permitted CHWs to improve mastery of procedures and appropriately perform participants’ inclusions and education activities regarding malaria. Similar results have been shown in previous experiences among the same community and intervention patterns during the Malakit project by Galindo et al. who concluded that practical training in the field was the most appropriate learning method for CHWs (39). In addition, a scoping review describing training, supervision, and quality of care in community-based programs in different contexts also concluded that training in the field and on-site improves CHW’s practice (51).

During the training, the differences among participants enhanced teamwork and learning from peers. In addition, differences allowed knowledge and experience sharing. Thus, they can be considered as a lever. Nevertheless, they added a level of complexity to training implementation.

Another important element to address is team building, which has been found out as a crucial element for the learning process and daily work in the field. During the training, the team-building activities helped to motivate CHWs and encouraged peer learning. Moreover, the sense of unity jointly with the engagement toward malaria elimination increased CHWs’ motivation in the daily working context, particularly in regions where malaria is in the process of being eliminated. The literature reports similar results, showing that relationships with other cadres and actors could play a major role in sustaining motivation (52, 53).

### 4.2 Initial and continuing training: an indispensable continuum

ToT is a good predictor of the long-term sustainability of public health initiatives (24). This ToT program was the starting point of a continuous professional development scheme; indeed, we considered continuing re-training as important as initial training. In fact, despite a widespread lack of ongoing training or other forms of continuing professional development, several studies have found a positive association between training and maintaining good standards of practice (54–57). This is shown to be linked (i) to refresh of acquired skills and knowledge and (ii) to motivation. For instance, Curtale et al. suggest that even a few days of additional training/supervision, if regularly provided, result in improved quality of service (58). Furthermore, access to training and supervision seems to be associated with non-monetary incentives sustaining CHWs’ motivation and engagement (13, 57). Our results showed the need for more time and fieldwork to master the step-by-step process and re-elaborate concepts for an effective knowledge transfer to the community, that is aligned with the literature.

A continuing training centered on CHWs seems to be an asset to the strategy of continuous professional development. Puchalski Ritchie et al., in their training course, established training contents on needs they identified through a prior qualitative survey performed by CHWs (59). CHWs interviewed for the training evaluation declared that constructive professional relationships, good teamwork, and a sense of belonging to a common cause were also pointed out by CHWs as factors able to influence behavior in the field, leading to engagement and proactive participation in the intervention. These recommendations together with our results sustain the design we adopted, particularly the bidirectional space we built to make the training program CHWs-centered.

Ongoing training, including various moments of evaluation and reflection, enables interventions to be adjusted in time to ensure proper implementation. Constant efforts throughout the project are essential to maintain quality while adapting to the inevitable changes in the context in which the intervention evolves.

### 4.3 Evaluation: a strength of training program implementation

In our experience, training evaluation has been done in the meantime of the training program. Consequently, the evaluation provided insights allowing adaptation during all training programs. Moreover, adaptations have been made to ensure better training from the initial training to the on-the-job one and address lacks as reported by CHWs interviewed. Evaluation provided useful information for trainers, allowing them to ensure a high-quality training program.

In current times, scientists call for studies examining the effectiveness and characteristics of training programs for CHWs to provide reliable evidence, avoid replication of programming difficulties, ensure high-quality training programs, and make possible standardization on the evaluation (60–64). Nevertheless, a limited number of studies exists regarding quality and effectiveness evaluation of training programs, especially regarding infectious diseases in cross-border areas and addressed to CHWs. Indeed, a recent review of the literature analyzed 62 articles on the evaluation of ToT and found only 5 in the field of infectious diseases and cross-border contexts, with heterogeneous and non-standardized methodologies (25). Reviewing articles on CHW-based programs for cardiovascular disease management in low-income and middle-income countries with the aim of evaluating training program effectiveness, Abdel-All and al. found only eight articles reporting training details on evaluation, among which 90 CHWs were eligible for their systematic review (60). This study answered this call and is aligned to the current scientific questions in this field.

Although all methodologies used in our evaluation are already well known and used on training evaluations, they are often not used together (25). Thus, our study provides a whole of mix methods for evaluation enabling triangulation and in-depth considerations. This study shows that developing a methodology for training evaluation is possible, feasible, and necessary to ensure advancements in this field. The same methodology could be re-used in our context and also transfer to similar training for CHWs.

These represent the major strengths of this study and suggest that the evaluation of training programs should be integrated into all CHW-based health interventions.

### 4.4 Limits of our study

The evaluation was carried out by researchers previously involved in the design, development, and implementation of the training. Although this evaluation design could lead to attribution bias, several data sources—both quantitative and qualitative—were used for the evaluation, enabling data triangulation and increasing internal validity.

Evaluation step 1 was conducted among both CHWs from Brazil and Suriname; on the contrary, only CHWs from Suriname (*n* = 4) participated in evaluation step 2. This was due to external factors linked to the Curema project implementation in the field, particularly since the Curema project has been progressively launched in the field starting from inclusion sites located in Suriname to finish with Brazilians. This could have resulted in poor in-depth inferences and points of view from CHWs from Brazil.

Kirkpatrick’s theoretical framework used recommends assessing effectiveness at four levels: reaction, learning, behavior, and system. The higher the impact in terms of effectiveness, the better the achieved final training outcome (25). Nevertheless, the assessment at the system level is more challenging, and we were unable to evaluate it. A further evaluation of learning transfer to the community at the end of the intervention could be realized by the post-intervention cross-sectional survey and would be very useful.

## 5 Conclusion

This study offers a practical example of the quality and efficacity evaluation of a ToT program for CHWs. The importance of appropriateness of training design including adult learning principles for the training program implementation was pointed out as essential to ensure efficacity of the training. Learning from peers and adaptation during the program also improved training efficacity. The training program strength was practical on-the-job training and continuous training as an indispensable part of supervision and continuous professional development. Goals included enhancing motivation among CHWs and promoting team and capacity building.

## Data availability statement

The raw data supporting the conclusions of this article will be made available by the authors, without undue reservation.

## Author contributions

CC: Conceptualization, Formal analysis, Methodology, Writing – original draft. IM: Conceptualization, Methodology, Writing – review & editing. MG: Conceptualization, Writing – review & editing. LP: Writing – review & editing. YL: Writing – review & editing. TB: Writing – review & editing. SV: Writing – review & editing. MS-M: Writing – review & editing. JB: Writing – review & editing. MD: Conceptualization, Writing – review & editing. AS: Conceptualization, Writing – review & editing.
